# Robotic Abdominal Surgery and COVID-19: A Systematic Review of Published Literature and Peer-Reviewed Guidelines during the SARS-CoV-2 Pandemic

**DOI:** 10.3390/jcm11112957

**Published:** 2022-05-24

**Authors:** Christina A. Fleming, Anna Fullard, Stefanie Croghan, Gianluca Pellino, Francesco Pata

**Affiliations:** 1 Department of Colorectal Surgery, University Hospital Limerick, V94 F858 Limerick, Ireland; 2 PROGRESS Fellow, Royal College of Surgeons in Ireland, D02 YN77 Dublin, Ireland; 3 Department of Colorectal Surgery, University Hospital Galway, H91 YR71 Galway, Ireland; anna.fullard@gmail.com; 4 Strategic Academic Recruitment (StAR) Programme (Urology) Royal College of Surgeons, D02 YN77 Dublin, Ireland; croghans@tcd.ie; 5 Department of Advanced Medical and Surgical Sciences, Universitá degli Studi della Campania “Luigi Vanvitelli”, 80138 Naples, Italy; gipe1984@gmail.com; 6 Colorectal Surgery, Vall d’Hebron University Hospital, 08035 Barcelona, Spain; 7 General Surgery Unit, Nicola Giannettasio Hospital, 87064 Corigliano-Rossano, Italy; francesco.pata@gmail.com; 8 La Sapienza University, 00185 Rome, Italy

**Keywords:** robotic surgery, SARS-CoV-2, COVID-19, pandemic, guidelines, abdominal surgery, outbreak

## Abstract

**Background:** Significant concern emerged at the beginning of the SARS-CoV-2 pandemic regarding the safety and practicality of robotic-assisted surgery (RAS). We aimed to review reported surgical practice and peer-reviewed published review recommendations and guidelines relating to RAS during the pandemic. **Methods:** A systematic review was performed in keeping with PRISMA guidelines. This study was registered on Open Science Framework. Databases were searched using the following search terms: ‘robotic surgery’, ‘robotics’, ‘COVID-19’, and ‘SARS-CoV-2’. Firstly, articles describing any outcome from or reference to robotic surgery during the COVID-19/SARS-CoV-2 pandemic were considered for inclusion. Guidelines or review articles that outlined recommendations were included if published in a peer-reviewed journal and incorporating direct reference to RAS practice during the pandemic. The ROBINS-I (Risk of Bias in Non-Randomised Studies of Intervention) tool was used to assess the quality of surgical practice articles and guidelines and recommendation publications were assessed using the AGREE-II reporting tool. Publication trends, median time from submission to acceptance were reported along with clinical outcomes and practice recommendations. **Results:** Twenty-nine articles were included: 15 reporting RAS practice and 14 comprising peer-reviewed guidelines or review recommendations related to RAS during the pandemic, with multiple specialities (i.e., urology, colorectal, digestive surgery, and general minimally invasive surgery) covered. Included articles were published April 2020—December 2021, and the median interval from first submission to acceptance was 92 days. All surgical practice studies scored ‘low’ or ‘moderate’ risk of bias on the ROBINS-I assessment. All guidelines and recommendations scored ‘moderately well’ on the AGREE-II assessment; however, all underperformed in the domain of public and patient involvement. Overall, there were no increases in perioperative complication rates or mortalities in patients who underwent RAS compared to that expected in non-COVID practice. RAS was deemed safe, with recommendations for mitigation of risk of viral transmission. **Conclusions:** Continuation of RAS was feasible and safe during the SARS-CoV-2 pandemic where resources permitted. Post-pandemic reflections upon published robotic data and publication patterns allows us to better prepare for future events and to enhance urgent guideline design processes.

## 1. Introduction

As we emerge from the devastating waves of the SARS-CoV-2 pandemic and enter a phase of recovery, it is opportune to reflect upon lessons learned from COVID-19. During the early phases of the pandemic, the necessity of continuing the delivery of safe surgery, in the context of rapidly increasing demands on hospital services and uncertainties regarding mechanisms of viral transmission, emerged as a major challenge [1,2,3,4]. In response, appropriately rapid communication of recommendations and guidelines emerged from regional, national, and local bodies. Such publications were widely disseminated and updated regularly to reflect relevant public health guidelines. To facilitate this, changes to publication practices were observed. COVID-related research was prioritised, with shorter than usual peer-review to publication times and an increase in open access dissemination [5]. This approach helped greatly to develop surgical practice and to protect patients, staff, and resources, whilst allowing further evolution as contemporaneous data emerged. However, perhaps unsurprisingly, some rapidly published findings were subsequently challenged and ultimately retracted.

Particular debate arose early in the pandemic in relation to the practice of minimally invasive surgery (MIS). Alongside obvious concerns regarding endotracheal intubation for general anaesthesia in the setting of a global severe respiratory pandemic, several unique aspects of MIS were questioned. Concerns were expressed that not all methods of viral transmission were fully understood and that positive pressure pneumoperitoneum and smoke plume by high-energy devices in laparoscopy and robotic-assisted surgery (RAS) could potentially lead to aerosolisation and transmission of SARS-CoV-2 viral particles [1,2]. However, as time progressed, MIS was confirmed to be safe, and potentially advantageous. It emerged that RAS may, in fact, confer greater benefit than risk in the context of a viral pandemic, by increasing the surgeon–patient interface via console operating, by reducing morbidity and facilitating shorter length of hospital stay (LOS) [6,7]. As the expected longevity of the pandemic became more apparent, the requirement for a sustainable approach to delivery of MIS was recognised. More recent literature and published recommendations highlight potential processes for ‘safe-site’, ‘clean-site’, and ‘COVID-free’ hospital pathways in which to deliver MIS, particularly in the context of time-sensitive surgical procedures.

Given this recent experience of a requirement for rapid acquisition and dissemination of data in a global healthcare crisis, unprecedented in the technological era, it is important to review our approach. Such reflection is desirable in order to assimilate learning points and attempt to ‘future-proof’ both our strategy for urgent guideline implementation and RAS itself. The aim of this review was to systematically evaluate studies referencing robotic surgery during the COVID-19 pandemic and to synthesise publication patterns, study design, outcome analysis, and reported clinical impacts of SARS-CoV-2 on RAS. A quality analysis of published peer-reviewed guidelines and review-based recommendations for the peri-pandemic practice of robotic surgery was also performed.

## 2. Methods

This systematic review and guideline appraisal was performed in adherence with PRISMA guidelines [8]. This review was prospectively registered in *Open Science Framework*, (https://osf.io/taz42, accessed on 3 February 2022), developed by the Center for Open Science (COS) to encourage integrity and reproducibility in research across scientific disciplines.

### 2.1. Search Strategy

A systematic and comprehensive literature search was performed using MEDLINE (PubMed), Embase (OvidSP), and the Cochrane Central Register of Controlled Trials (CENTRAL) in the Cochrane Library. The following search and MeSH (medical subject headings) terms and free-text were used: ‘robotic surgery’, ‘robotics’, ‘COVID-19’, and ‘SARS-CoV-2’. The full search string is available in Supplementary Materials Data S1. Following this search, duplicate articles were identified and removed, and all remaining identified citations were screened for inclusion suitability. Title and abstracts were screened and potentially suitable articles subsequently included in the full text review. Reference lists of eligible studies were also searched to identify any additional relevant studies. Studies published in the English language were eligible. Studies published from 1 January 2020 to 1 January 2022 were identified and the latest search performed on 4 February 2022.

### 2.2. Inclusion and Exclusion Criteria

Peer-reviewed publications reporting on robotic surgery and COVID-19 or the SARS-CoV-2 pandemic were considered for inclusion. Original articles describing any outcome from or making reference to robotic surgery during the COVD-19/SARS-CoV-2 pandemic were eligible for inclusion as were peer-reviewed publications providing guidelines or recommendations for practice. The following article types were excluded: review articles where recommendations or guidelines were not presented, case reports reporting <five cases, letters to the editor, and commentary pieces. Where an abstract and final manuscript representing the same work were both identified, the abstract was excluded and data were extracted from the final manuscript. Articles were excluded if they did not reference the specific impact of the pandemic on robotic surgery. Guidelines or review articles outlining recommendations were included if published in a peer-reviewed journal and making direct reference to RAS practice during the pandemic. Specialty association and national and international guidelines that were website-published were excluded due to the fact of their dynamic nature; however, it was highlighted and recorded where each of these guidelines were referenced in published articles. Articles relating to robotic-assisted nonsurgical procedures (e.g., endoscopy and percutaneous coronary interventions) were excluded. Video vignettes were also excluded. At each stage, the reason for study exclusion was documented, and disagreement on suitability of article inclusion was resolved by discussion between three authors (C.A.F., A.F., and S.C.) until consensus was reached.

### 2.3. Quality Assessment

Quality assessment of the methodological design and delivery of included studies was assessed using the Cochrane Risk of Bias Tool for randomised controlled trials (RCTs) and ROBINS-I (Risk of Bias in Non-Randomised Studies of Intervention) tool for all nonrandomised studies as appropriate [9,10]. Guideline and recommendation publications were assessed using the AGREE-II reporting tool, an internationally accepted standard for evaluation of the methodological quality of clinical practice guidelines [11]. The AGREE-II assessment tool results in a cumulative score (out of a possible maximum of 161) across six domains: 1—scope and purpose (21); 2—stakeholder involvement (21); 3—rigour of development (56); 4—clarity of presentation (21); 5—applicability (28); 6—editorial independence (14).

### 2.4. Outcomes of Interest

For studies reporting on RAS practice during the COVID-19 pandemic, outcomes of interest included overall study aim, study design, clinical and service outcomes of interest in the specific context of robotic surgery, and main study findings, again specifically relating to robotic surgery. For guideline and review recommendation articles, further outcomes of interest included overall aim; nonpeer-reviewed guidelines included basis of recommendations and conclusions relevant to RAS. For both article types, publication timeframes were reviewed, including month and year of publication and interval from first submission to acceptance of articles for publication.

### 2.5. Data Extraction, Synthesis, and Analysis

Basic study characteristic data were extracted including the following: name of first author, year of publication, study duration, and location of study conduct. Study aim, study design outcomes, and reported impact of COVD-19 in the context of robotic surgery practice and service delivery were also extracted. Overall the aims and recommendations of included guidelines were also extracted. Required data to perform a risk of bias and AGREE-II assessment were also extracted.

A narrative synthesis was performed for study characteristics, aims, and outcomes. The quality of guidelines and recommendations using AGREE-II tool scores were reported on continuous scales as overall scores and categorically within the six included domains, with median and interquartile range (IQR) calculated using Excel (Microsoft 2016).

## 3. Results

### 3.1. Search Results

**Figure 1:**
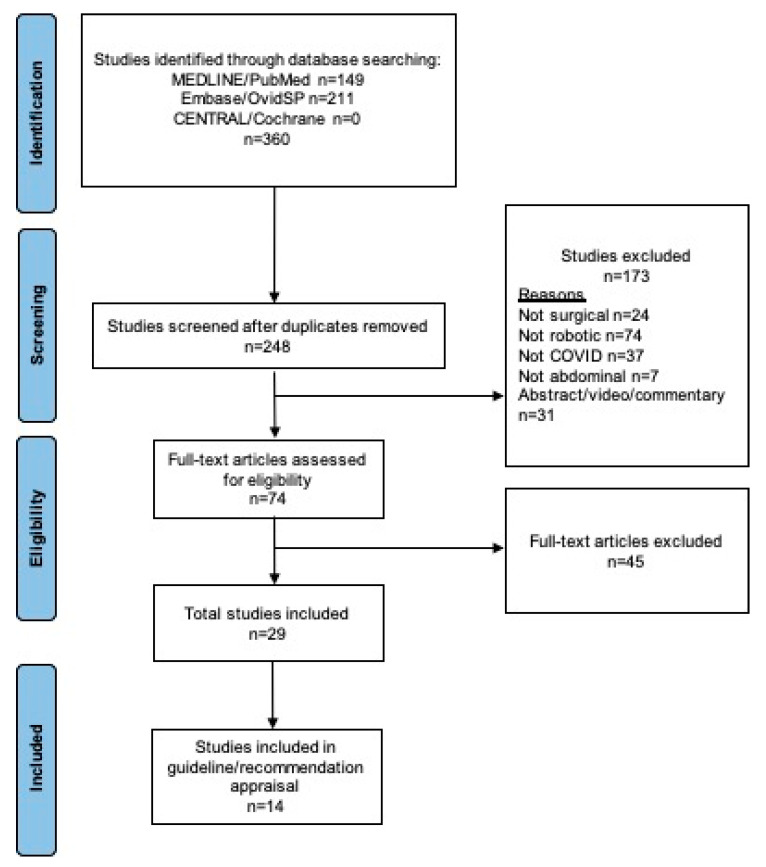
PRISMA flowchart of study assessment, exclusion (including reasons), and inclusion.

The initial literature search identified 360 articles. Following removal of duplicates, a total of 248 articles were identified, of which 29 were deemed suitable for final inclusion (Figure 1). Of articles ultimately included, 15 related to studies of RAS practice during COVID-19, and 14 were guidelines or review recommendations.

### 3.2. Quality Assessment and AGREE-II

Studies reporting RAS practice during the COVID-19 pandemic were all nonrandomised clinical studies and, thus, all quality assessments performed using ROBINS-I (Supplementary Materials Table S1). Three studies were deemed to be at moderate risk of bias [12,13,14] and the other 12 were deemed at low risk of bias (Table 1). Two studies were deemed to be of low risk of bias across all domains [15,16] and one study deemed moderate risk across all domains [13]. The domain most frequently scoring at moderate risk of bias was “measurement of outcome” (moderate risk in 75%, n = 10 studies).

**Figure 2:**
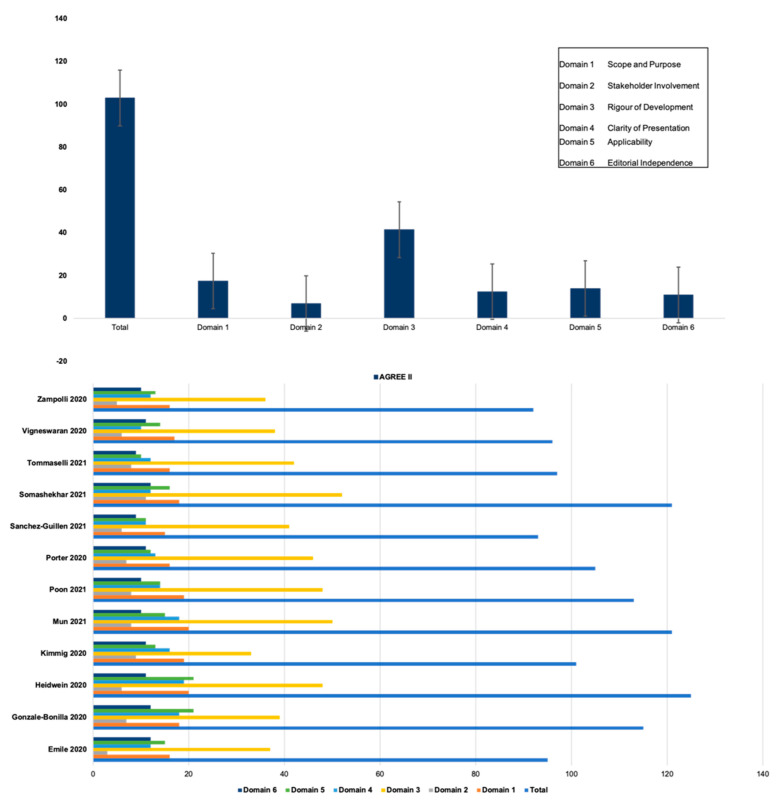
AGREE-II Comparison.

AGREE-II assessment scores are summarised in Table 2 and Figure 2 The overall median AGREE-II score was 105 (IQR: 17). All articles performed poorly in domain two, ‘stakeholder involvement’ (median: 7, IQR: 6). This was, in all cases, due to the inadequacies in public and patient involvement, alongside high stakeholder involvement from the perioperative team. All studies scored highly in domain three, ‘rigour of development’ (median: 42, IQR: 9), and domain one, ‘scope and purpose’ (median: 18, IQR: 3).

### 3.3. Publication Trends

**Figure 3:**
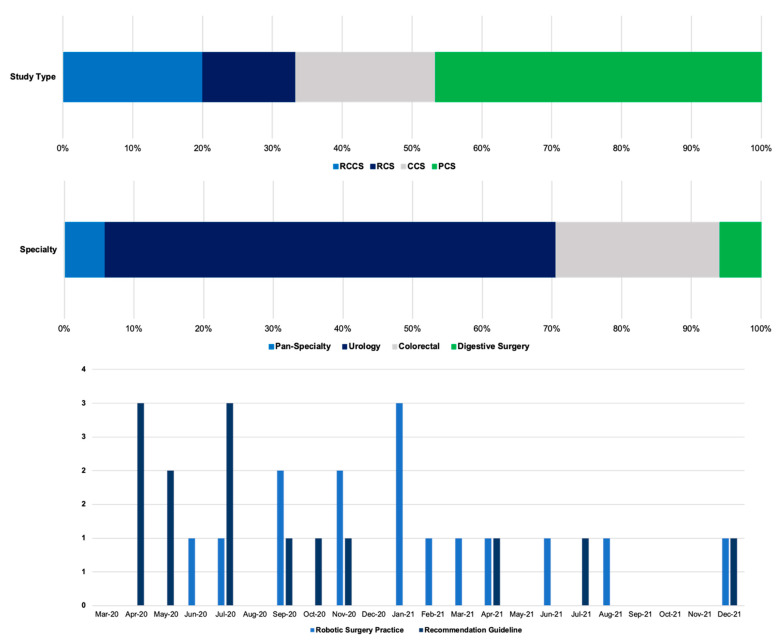
Study type and relevant surgical specialty for publications related to robotic-assisted surgery practice during the COVID-19 pandemic and publication patterns of both robotic surgery practice and peer-reviewed recommendation/guideline articles.

All included articles were published between April 2020 and December 2021 (Figure 3). The highest volume was published in in January 2021 (n = 3), followed by September and November 2020 (both n = 2). The highest volume of guidelines/recommendations were published in April 2020 and July 2020 (both n = 3). The interval from first submission to acceptance was available for 14 of 15 RAS surgical practice articles, with a calculated median interval of 92 days. The interval to acceptance for guidelines or review recommendations was available in nine of 14 articles, with a median interval of five days.

### 3.4. Robotic Surgery Practice during COVID-19 Pandemic

Fifteen articles reporting on the practice of RAS during the COVID-19 pandemic were suitable for inclusion (Table 1), reporting on data from 5193 patients [6,7,12,13,14,15,16,17,18,19,20,21,23,24]. This included seven prospective cohort studies (PCS), three cross-sectional cohort studies (CCS), three retrospective comparative cohort studies (RCCS), and two retrospective cohort studies (RCS). Articles related to RAS practice in the setting of urological surgery (n = 10), colorectal surgery (n = 3), digestive surgery (n = 1), pan-specialty practice (n = 1), and combined urology/colorectal practice (n = 1).

The primary aim of studies varied and included investigation of the safety of continuing same-day discharge following urological RAS (n = 1), of the impact of COVID-19 on RAS volume (n = 4), of resident training in RAS during the pandemic (n = 2), of “clean-site” clinical pathways for RAS during the COVID-19 pandemic and of patient outcomes following RAS during COVID-19 (n = 6). As expected, overall reduction was observed in the volume of RAS practiced. The extent of this reduction, however, varied and some centres maintained an RAS volume of approximately 50% of pre-pandemic levels, especially where clinical pathways were in place to facilitate robot-assisted surgery. Overall, no increase in perioperative complication rates or mortality was identified in patients who underwent RAS during the COVID-19 pandemic compared to that expected baseline pre-pandemic rates or pandemic open rates. In the reported perioperative COVID-19+ cases in these studies of RAS, one post-operative mortality was reported in the literature in a COVID+ patient post robotic-assisted radical prostatectomy (RARP).

### 3.5. Guidelines and Review Recommendations

Fourteen articles reported a clinical guideline or review paper with recommendations for the practice of RAS during COVID-19 pandemic (Table 2) [25,26,27,28,29,30,31,32,33,34,35]. Six articles were produced as general MIS recommendations/guidelines, and eight were specialty-specific (n = 3 urology; n = 2 colorectal; n = 1 (each) digestive, gynaecology, and metabolic surgery). Providing guidance on the overall safety of MIS during COVID-19 was the main aim of the article in 57% (n = 8), whilst 29% (n = 4) were specific to speciality practice. All except one guideline/review paper referenced non-peer-reviewed guidelines from surgical specialty organisations, most commonly the Society of American Gastrointestinal and Endoscopic Surgeons (SAGES), the European Association of Endoscopic Surgery (EAES), and the European Association of Urology (EAU) [1,2,3].

The overall recommendations from these peer-reviewed, published articles were supportive of the continued practice of RAS during the COVID-19 pandemic. One article recommended that RAS should be limited to urgent and time-sensitive procedures only. This was published in May 2020, within the initial lockdown period. Most studies recognised the potential benefit of RAS during a global pandemic where viral transmission may occur via aerosolisation due to the increased distance existing between the surgeon and patient with console operating. All safety guidelines recommended clear steps to optimise the safety of MIS and RAS during COVID-19, including, but not limited to, maintenance of operating room staff to the minimum number required, provision of appropriate personal protective equipment (PPE) for all staff members, minimisation of positive pressure pneumoperitoneum time and pressures, and utilisation of ultralow particulate air filters where available. Pre-operative SARS-CoV-2 screening of patients for elective surgery was also recommended.

## 4. Discussion

In this review, we report on 29 articles, including 15 that present findings on RAS practice and related outcomes during the SARS-CoV-2 pandemic, and 14 that present RAS practice recommendations for the COVID-19 era. A combination of prospective and retrospective studies was reported across multiple surgical specialties. Overall, the practice of RAS emerged as safe, with no increase in perioperative complication rates or mortalities in patients who underwent RAS during the pandemic compared to quoted incidences based on non-COVID practice. In general, published guideline and review recommendations also supported continued practice of RAS, whilst making suggestions for mitigation of potential risk of viral transmission.

The practice of robot-assisted surgery experienced a tumultuous course during the pandemic. Initially, assumptions of an increased risk of aerosolisation and transmission of viral particles secondary to pneumoperitoneum and smoke plume creation prevailed, causing laparoscopic and robotic procedures to be disfavoured [1,2,3]. In the ACIE Appy study [39], focused on the management of acute appendicitis in 2020, one-third of the 709 responders declared a shift toward open surgery as a consequence of advice from expert groups and surgical societies in the early phases of the pandemic [40,41,42,43].

In addition, RAS was considered by many to represent an inequitable use of resources in the context of unprecedented demand, shortfall, and rationing of operating theatre availability, equipment, and personnel. Accordingly, early recommendations suggested that open surgery should be chosen over MIS during the COVID-19 pandemic. As a result, RAS practice dramatically reduced in the early phase of the pandemic, with Da Vinci surgical system (Intuitive Surgical, Sunnyvale, CA, USA) weekly usage figures from March 2020 demonstrating a 65% decrease relative to those earlier in the same quarter [44].

Despite these early concerns regarding RAS in the setting of the COVID-19 pandemic, the evidence did not identify inferior clinical outcomes related to RAS practice during the pandemic [7,16,19,21,23]. In fact, some benefits associated with utilisation of a robotic-assisted approach to surgery during the pandemic were identified. These included the creation of increased distance between the operating surgeon and patient, potentially reducing the risk of pathogen contamination, enabling faster patient recovery with consequent decreased length of stay and exposure to the hospital environment, and potential reduction in the requirement for post-operative intensive-care admission for certain procedures [45,46,47].

The guidelines and review-based recommendations summarised in this review pragmatically recognised the safety of RAS during the pandemic, whilst suggesting measures to mitigate risk of viral transmission [25,27,28,29,32,37,38]. This represented a shift from dynamic recommendations that were disseminated early in the pandemic. Clinical practice appears to reflect the adoption of these more recent guidelines. In the later stage of the pandemic, analysis of procedure volume and revenues of the dominant robotic platform supplier (i.e., Intuitive Corporation, Sunnyvale, CA, USA) showed maintenance of global procedure volume, albeit with blunting in expected growth, in the context of the pandemic (global procedure volume: 1.04 million in 2018, 1.23 million in 2019, and 1.24 million in 2020) [48].

The evolution of the COVID-19 pandemic created a pressing need for rapid creation and dissemination of information globally, as an unknown entity faced healthcare systems worldwide. Rapid availability of early data and guidance was critical for preparing health services for incoming waves of SARS-CoV-2 by facilitating information sharing and allowing lessons to be learnt from preliminary experiences of global colleagues [49,50]. This requirement, unprecedented in its urgency during the digital age, led to a high volume of non-peer-reviewed, web-based information and guidance appearing. Access to this information was essential at the time [5]. However, as we emerge from the pandemic, we are uniquely positioned to reflect upon information dissemination processes, to ensure that lessons are learned and that systems are optimised for potential future similar events. We need to ensure that robust mechanisms are in place for dynamic and rapid dissemination of information in a global pandemic but also ensure that we reduce or avoid dissemination of work that ultimately transpires to be inaccurate and requires retraction [51,52].

Encouragingly, assessment of the peer-reviewed guidelines and practice recommendations included in this review, by application of the AGREE-II tool, resulted in relatively high overall scores. One domain in which marked underperformance was apparent was that of public and patient involvement, which therefore stands out as an area for research focus and improvement in the instance of future global health crises. A further noteworthy factor was time from submission to acceptance for publication of such peer-reviewed documents. This was remarkably short, at a median of five days. Whilst rapid dissemination was, and always will be, highly desirable in such contexts, and applying minimum timeframes for article evaluation would likely be arbitrary and meaningless, it is imperative that stringent review processes are guaranteed and that individual publishing bodies ensure that quality control is upheld during both times of crises and times of relative normalcy.

There were a number of limitations to this work. Exclusively peer-reviewed published guidelines and recommendations were included, with resultant exclusion of some highly cited specialty association, national, and international guidelines that were website-published. This approach was given due consideration; however, it was ultimately not felt possible to include the latter publication types due to the fact of their dynamic nature. This meant that they were actively updated over time, and time-specific quality assessment was therefore prohibited. This review, did, however record and highlight the presence of such guidelines amongst the references of peer-reviewed articles. This work also focused on robotic-assisted surgery solely in the domain of abdominal and pelvic surgery. It is important to recognise that specific additional issues may be relevant to the peri-pandemic conduct of RAS in other bodily cavities, for example, in otolaryngology and oral and maxillofacial surgery.

Numerous lessons have been learnt from the experience of RAS practice in the context of a respiratory pandemic, and the international surgical community must capitalise upon these in the unfortunate instance of a future similar events. Knowledge gained on the safety of RAS from an aerosolisation perspective during COVID-19 will markedly allay concerns and uncertainties if faced with subsequent pandemics characterised by airborne-transmission of viral particles and, hence, facilitate smoother continuation of service provision. The possibility of rapid peer-review and dissemination of data and guidelines has been highlighted, setting unprecedented standards for speed of research communication that should be aspired to in future crises. However, potential weaknesses associated with publishing in a climate where velocity is paramount have been uncovered. In accordance with these, future pandemic-based research should focus on strategic mechanisms to ensure patient and public involvement without causing undue delays, and researchers, peer-reviewers, editors, and publishers must work in tandem to ensure the highest quality of review processes are implemented, resulting in recommendations that are both rapidly communicated and scientifically robust.

## 5. Conclusions

Continuation of robotic-assisted abdominal and pelvic surgery was confirmed to be feasible and safe during the SARS-CoV-2 pandemic, where resources permitted its continued practice. In the aftermath of the pandemic, reflections upon both published data pertaining to RAS and peri-pandemic publication patterns allow us to create healthcare systems that demonstrate enhanced preparedness for future events, and publication systems adept at briskly designing, producing, and disseminating scientifically robust and widely generalizable guidelines and recommendations in the face of rapidly evolving global health crises.

## Figures and Tables

**Table 1:** Study and publication characteristics of studies reporting on robotic surgery during the SARS-CoV-2 pandemic.

Author	Publication Date	Interval to Publication	Specialty	Country	No. Patients	Study Design	Study Timing	Aim	Outcomes	Main Finding	ROBINS-I
**Abaza** **2021** [6]	January 2021	70	Urology	USA	131	RCCS	16 March to 5 June 2020	Impact of COVID-19 on SDD following RAS urologic surgery	30 day morbidity Perioperative pain scores Pathological outcomes	SDD was safely applied during COVID-19 pandemic without increasing complications or readmissions	Low
**Abou-Chedid** **2021** [12]	December 2021	241	Urology	UK	1998	RCCS	23 March to 10 May 2020	Impact of COVID-19 on RAS surgical volume in urologic oncology	Impact of COVID-19 on RAS surgical training in urologic oncology	Substantial decrease in RAS urological oncology caseload during the COVID pandemic (clean sites can ensure continuity of care for cancer surgery and training needs)	Moderate
**Blanc** **2021** [17]	January 2021	23	Pan-specialty	France	535	RCCS	16 March to 30 April 2020	Assess impact of COVID-19 pandemic on RAS volume	Perioperative COVID status COVID management	60% decreases in RAS volume 49% decrease in RAS oncological procedures	Low
**Busetto** **2020** [18]	November 2020	151	Urology	Italy	387 Residents (RR 67.1%)	CCS	March to April 2020	Identify positive changes to urology residency due to COVID-19 pandemic	Impact of pandemic on training volume Role of social distancing distance learning and telemedicine	52.9% used distance teaching for the first time Working in COVID hospital significantly reduced RAS activity (OR 4.64)	Low
**Evans** **2020** [15]	July 2020	3	Colorectal	UK	38	PCS	25 March to 9 April 2020	Early experience of colorectal cancer surgery clinical pathway during COVID-19 pandemic	30 day morbidity LOS Pathological outcomes	78% MIS rate (17% RAS) No major post-operative complications No perioperative diagnosis of COVID-19	Low
**Harke** **2020** [14]	September 2020	61	Urology	Germany	27 Urology centres (RR 41%)	CCS	16 March to 24 May	Changes in urologic practice during the COVID-19 pandemic with particular focus on robotic surgery	Surgeon reported changes in overall capacity, elective/emergency surgery volume, and perceived protection of a robotic surgery approach	27% reduction in robotic surgery Equal reductions in open and robotic procedures	Moderate
**Huddy** **2021** [7]	February 2021	103	Colorectal and Urology	UK	60	PCS	12 May to 30 July 2020	Experience at a “COVID protected” robotic surgical centre during the COVID-19 pandemic	Operative time Blood loss Complications Readmissions	Safe delivery of robotic surgery in dedicated unit with acceptable outcomes Reduced LOS due to the dedicated unit	Low
**Minervini** **2021** [19]	April 2021	92	Urology	Italy	1943	PCS	24 February to 30 March 2020	Surgical outcomes in patients undergoing elective urological surgery during the COVID-19 pandemic	Operative practice trends Perioperative outcomes COVID+ rates and related outcomes	21.3% of cases were performed robotically One post-operative mortality in robotic cohort due to the fact of pneumonia (SARS-CoV-2 PCR positive)	Low
**Moschovas** **2021** [20]	November 2020	-	Urology	USA and Italy	585	PCS	1 March to 25 May 2020	Management of patients with prostate cancer during the COVID-19 pandemic	Management algorithm Selection criteria for surgery Perioperative management	147 RARRPs performed without complications	Low
**Motterle** **2020** [16]	June 2020	6	Urology	Italy	77	PCS	9 March to 1 May 2020	MIS urology practice during the COVID-19 “lockdown” period	Practice trends SARS-CoV-2 testing, PPE and aerosolisation reduction strategies	80.5% of cases performed RAS 7.8% 15 day complication CD > 3 No perioperative patient/staff COVID positive	Low
Ö**zdemir** **2021** [21]	August 2021	2	Digestive Surgery	Turkey	129	PCS	March 2020 to May 2021	Surgical management of gastrointestinal tumours in a COVID-19 pandemic hospital	List of procedures Operation time LOS Pathological outcomes	13.2% performed robotic 8 cases of perioperative COVID+, no mortalities	Low
**Sobrado 2021** [22]	March 2021	96	Colorectal	Brazil	103	RCS	10 March to 9 Sept 2020	Safety of elective colorectal surgery during the COVID-19 pandemic	Variety of procedures performed Perioperative outcomes Perioperative COVID rates	90.9% colorectal cancer 9.1% IBD 3% performed RAS	Low
**Sparwasser** **2021** [23]	June 2021	65	Urology	Germany	61	RCS	12 March to 11 May 2020	Investigate the safety of RAS during the COVID-19 pandemic concerning newly acquired COVID rates	Perioperative outcomes Perioperative COVID+ rates	11.5% >/= CD Grade III complication 1.6% (n = 1) post-operative COVDI+	Low
**Tabourin** **2020** [24]	September 2020	103	Urology	France	68	PCS	2 March to 14 April 2020	To assess potential COVID-19 rates in RAS procedures	Perioperative outcomes Perioperative COVID+ rates	91.8% RAS procedures for oncology 16.2% symptomatic post-op and 1.5% (n = 1) tested positive	Low
**Teixeira** **2021** [13]	January 2021	105	Urology	Portugal	43 Residents (RR 54.4%)	CCS	25 April to 25 May 2020	Impact of the COVID-19 pandemic on urology residence in Portugal	Impact on operative activity Impact on case-mi xImpact on clinical activities and emergency practice	34.9% RAS postponed RAS volume reduced by 65.9%	Moderate

CCS, cross-sectional cohort study; LOS, length of hospital stay; MIS, minimally invasive surgery; PCS, prospective cohort study; PPE, personal protection equipment; SDD, same-day discharge; RAS, robotic-assisted surgery; RCS, retrospective cohort study; RCCS: retrospective comparative cohort study; RR, response rate.

**Table 2:** Study and publication characteristics of peer-reviewed, published recommendations and guidelines related to robotic surgery during the SARS-CoV-2 pandemic.

Author	Specialty	Country	Publication (Month, Year)	Publication Interval (Days)	Overall Aim	Non-Peer-Reviewed Guidelines Included	Basis of Recommendation	Conclusions Relevant to Robotic Surgery	AGREE II Score (/161)
**Emile** **2020** [25]	Digestive Surgery	International	October 2020	84	Safety of MIS (including robotic surgery) amid the COVID-19 pandemic	ACS EAES RCSEd SAGES	Evidence based	Robotic surgery is safe to continue in abdominal Emergency Caution should be taken to avoid the presumed risk of aerosolisation of the virus particles during procedures	95
**Gallo** **2020** [26]	Colorectal Surgery	Italy	April 2020	1	Provide national good clinical practice guidelines during the COVID-19 pandemic	ACPGBI ACS ECCO SAGES	Mixed-evidence based and expert consensus	Potential hazards of robotic surgery need to be weighed against the benefits (shorter length of stay and decreased complication rate)	113
**Gonzalez-Bonilla** **2020** [27]	Urology	Mexico	June 2020	5	Evidence and recommendations for urologic-RAS during the COVID-19 pandemic	ACS ASA SAGES	Mixed-evidence based and expert consensus	RAS viable with proper precautions	115
**Heldwein 2020** [28]	Urology	International	June 2020	-	Summarise guidelines and recommendations on urology of care during the COVID-19 pandemic	EAU	Evidence based	RAS feasible with: -minimum number of OR staff -adequate PPE -avoid positive pressurisation	125
**Kimmig 2020** [29]	Gynaecology (SERGS)	International	April 2020	1	Provide guidance for gynaecology surgery during the COVID-19 pandemic	ACS EAES EAU RCOG SAGES	Mixed-evidence based and expert consensus	RAS feasible Technical recommendations to reduce aerosolisation during RAS	101
**Mun 2021** [30]	MIS-Abdominal	International	September 2020	-	Evaluate guidelines and clinical activity regarding aerosolisation risk during the COVID-19 pandemic	ACS EAES EAU RCS SAGES	Mixed-evidence based and expert consensus	RAS feasible Lack of evidence that RAS increases risk of viral transmission	121
**Navarra 2020** [31]	Metabolic Surgery	Italy	June 2020	3	Recommendations for triage of surgical procedures in morbidly obese patients during the COVID-19 pandemic	ACS EAES SAGES	Mixed-evidence based and expert consensus	RAS allows for staff and surgeons to be remote from patient Use of ultralow particulate air filters encouraged	105
**Poon 2021** [32]	Urology	China	April 2021	-	Prostate cancer management recommendations	EAU	Mixed-evidence based and expert consensus	Screen patients for SARS-CoV-2 prior to RAS procedures Exercise caution regarding aerosolisation	113
**Porter 2020** [33]	MIS	International	May 2020	-	Recommendations to mitigate COVID-19 transmission during MIS	AAGL EAES EAU RCS RCSEd SAGES	Mixed-evidence based and expert consensus	Limit RAS to urgent/emergency procedures Mitigation of aerosolisation risk PPE and minimum OR staff	105
**Sanchez-Guillen 2021** [34]	Coloproctology	Spain	June 2021	135	Approach to robotic and transanal surgery peri-COVID-19 pandemic	ACPGBI EAES ESCP SAGES	Evidence based	RAS offers advantages, e.g., theatre staff safety (additional physical distance from patient) Risk reduction techniques should be exercised	101
**Somashekhar 2021** [35]	MIS	International	November 2020	67	Safety modifications to perform MIS during the COVID-19 pandemic	ACS EAES EAU SAGES	Evidence based	Low risk of transmission during RAS Barrier benefits to surgeons and OR staff Minimises aerosolisation	121
**Tommaselli 2021** [36]	MIS	USA	December 2021	-	Critically appraise recommendations on measures to reduce the risk of SARS-CoV-2 transmission to OR staff during MIS	ACS EAES EAU RCS RCSEd SAGES	Evidence based	RAS should be performed when needed Strategies to minimise aerosolisation	97
**Vigneswaran 2020** [37]	MIS	USA	April 2020	Same day	Evaluate safety and use of MIS during the COVID-19 pandemic	SAGES	Evidence based	RAS feasible Strategies to minimise aerosolisation	96
**Zampolli 2020** [38]	MIS	International	May 2020	15	Risk of viral transmission during MIS	-	Evidence based	RAS considered safe Modification of standard procedures to reduce aerosolisation	92

AAGL, American Association of Gynecologic Laparoscopists; ACPGBI, Association of Coloproctology in Great Britain and Ireland; ACS, American College of Surgeons; ASA, American Society of Anesthesiologists; EAES, European Association of Endoscopic Surgery; EAU, European Association of Urology; ECCO, European Crohn’s and Colitis Organisation; ESCP, European Society of Coloproctology; MIS, minimally invasive surgery; OR, operating room; PPE, personal protective equipment; SAGES, Society of American Gastrointestinal and Endoscopic Surgeons; RAS, robotic-assisted surgery; RCOG, Royal College of Obstetricians and Gynaecologists; RCS, Royal College of Surgeons; RCSEd, Royal College of Surgeons in Edinburgh.

## Data Availability

The data presented in this study are available upon request from the corresponding author.

## Supplementary Materials

The following supporting information can be downloaded at: https://www.mdpi.com/article/10.3390/jcm11112957/s1, Data S1: Search string; Table S1: ROBINS-I Risk of Bias Assessment for surgical practice of robotic surgery studies; Table S2: Completed scores following AGREE-II assessment of included guideline and recommendation articles with break down into each domain.
