# Supplementary material for: Robotic Abdominal Surgery and COVID-19: A Systematic Review of Published Literature and Peer-Reviewed Guidelines during the SARS-CoV-2 Pandemic
Source: J Clin Med. 2022 May 24;11(11):2957. doi: 10.3390/jcm11112957 (PMC9181746; doi:10.3390/jcm11112957)
Supplement: Supplementary file 1 [file jcm-11-02957-s001.zip › jcm-1689925-supplementary.pdf]

Supplementary Data

Supplementary Data S1: Search String

'robotic surgery' OR 'robotics' OR 'robotic' AND 'COVID-19' OR 'COVID' OR 'SARS-CoV-2'

Supplementary Table S1: ROBINS-I Risk of Bias assessment for surgical practice of robotic surgery studies

Study Author and Year	Overall Bias	Confounding Variables	Participant selection	Classification of interventions	Deviation from Intention to Treat	Incomplete Data	Measurement of Outcomes	Selective Reporting
Abaza 2021	Low	Low	Moderate	Low	Low	Low	Moderate	Low
Abou-Chedid 2021	Moderate	Moderate	Low	Moderate	Moderate	Moderate	Moderate	Moderate
Blanc 2021	Low	Low	Low	Moderate	Low	Low	Moderate	Low
Busetto 2020	Low	Low	Low	Moderate	Low	Low	Moderate	Low
Evans 2020	Low	Low	Low	Low	Low	Low	Low	Low
Harke 2020	Moderate	Moderate	Moderate	Low	Low	Low	Moderate	Low
Huddy 2021	Low	Low	Low	Low	Moderate	Low	Low	Low
Minerva 2021	Low	Low	Low	Moderate	Low	Low	Low	Low
Moschovas 2021	Low	Low	Low	Moderate	Low	Low	Low	Low
Motterle 2020	Low	Low	Low	Low	Low	Low	Low	Low
Özdemir 2021	Low	Low	Low	Low	Low	Low	Moderate	Low
Sobrado 2021	Low	Low	Low	Moderate	Low	Low	Moderate	Low
Sparwasser 2021	Low	Low	Low	Moderate	Low	Low	Moderate	Low
Tabourin 2020	Low	Low	Low	Moderate	Low	Low	Moderate	Moderate
Teixeira 2021	Moderate	Moderate	Moderate	Moderate	Moderate	Moderate	Moderate	Moderate

Low
Moderate
High
Not Reported

Supplementary Table S2: Completed scores following AGREE-II assessment of included guideline and recommendation articles with break down into each domain. A total score of 161 is possible with potential domain totals as follows: D1 21, D2 21, D3 56, D4 21, D5 28, D6 14.

Study	Domain 1: Scope and Purpose	Domain 2: Stakeholder Involvement	Domain 3: Rigour of Development	Domain 4 : Clarity of Presentation	Domain 5: Applicability	Domain 6: Editorial Independence	AGREE II Score (Total)
Emile 2020	16	3	37	12	15	12	95
Gallo 2020	17	6	42	18	19	11	113
Gonzale-Bonilla 2020	18	7	39	18	21	12	115
Heidwein 2020	20	6	48	19	21	11	125
Kimmig 2020	19	9	33	16	13	11	101
Mun 2021	20	8	50	18	15	10	121
Navarra 2020	19	6	39	17	13	11	105
Poon 2021	19	8	48	14	14	10	113
Porter 2020	16	7	46	13	12	11	105
Sanchez-Guillen 2021	15	8	41	17	11	9	101
Somashekhar 2021	18	11	52	12	16	12	121
Tommaselli 2021	16	8	42	12	10	9	97
Vigneswaran 2020	17	6	38	10	14	11	96
Zampolli 2020	16	5	36	12	13	10	92
